# A Novel Method Based on ERP and Brain Graph for the Simultaneous Assessment of Various Types of Attention

**DOI:** 10.1155/2022/6318916

**Published:** 2022-09-29

**Authors:** Ali Esmaili Jami, Mohammad Ali Khalilzadeh, Majid Ghoshuni, Mohammad Mahdi Khalilzadeh

**Affiliations:** Department of Biomedical Engineering, Mashhad Branch, Islamic Azad University, Mashhad, Iran

## Abstract

Assessment of attention is of great importance as one of human cognitive abilities. Although neuropsychological tests have been developed and used to evaluate the ability to pay attention, their validity and reliability have been reduced due to some limitations such as the presence of intervention factors, including human factors, limited range of languages, and cultural influences. Therefore, direct outputs of the brain system, represented by event-related potentials (ERPs), and the analysis of its function in cognitive activities have become very important as a complementary tool to assess various types of attention. This research tries to assess 4 types of attention including sustained, alternative, selective, and divided, using an integrated visual-auditory test and brain signals simultaneously. Thus, the electroencephalogram (EEG) data were recorded using 19 channels, and the integrated visual and auditory (IVA-AE) test was simultaneously performed on twenty-eight healthy volunteers including 22 male and 6 female subjects with the average age of 27 ± 5.3 years. Then ERPs related to auditory and visual stimuli with synchronous averaging technique were extracted. A topographic brain mapping (topo-map) was plotted for each frame of stimulation. Next, an optical flow method was implemented on different topo-maps to obtain motion vectors from one map to another. After obtaining the overall brain graph of an individual, some features were extracted and used as measures of local and global connectivity. The extracted features were consequently evaluated along with the parameters of the IVA test by support vector machine regression (SVM-R). The volume of attention was then quantified by combining the IVA parameters. Ultimately, estimation accuracy of each type of attention including focused attention (86.1%), sustained attention (83.4%), selective attention (80.9%), and divided attention (79.9%) was obtained. According to the present study, there is a significant relationship between response control and attention indicators of the IVA test as well as ERP brain signals.

## 1. Introduction

Attention refers to a series of complex mental operations, which includes engaging and focusing on a goal, holding or tolerating, and being alert for a long period as well as encoding stimulus properties and shifting focus from one target to another [1]. Attention plays a significant role in carrying out our daily activities and it is a key factor in the process of learning and memorizing [2]. There are some limitations in neuropsychological tests such as lack of tests with specific criteria to check the change in a person's performance over time, depending on the culture of the tests, the length of the tests not being able to be translated into other languages, and the lack of valid and predictive local tests [3]. Therefore, validity and reliability of the tests decrease due to limitations such as the existence of intervention factors, especially human factors. The use of direct outputs of the brain system, the analysis of its function in cognitive activities, and the creation of intelligent systems with the help of machine learning have become very important in health care.

In previous studies, brain signals have been utilized for two purposes to evaluate attention. In some articles, only the separation between the group of healthy people and those with attention disorder or ADHD by using brain signals and attention tests has been discussed [4, 5]. In some other articles, the estimation of one of the five types of attention during a specific activity by brain signals has been discussed [6, 7].

So far, various methodological approaches have been developed in order to measure different types of attention in combination with neuroimaging techniques. The mentioned methods include electroencephalography focusing on the measurement of event-related potentials (ERPs) for various purposes in the field of sports or various cognitive disorders, such as the study of sustained attention in combat sports athletes, attention-oriented processes in children with attention deficit hyperactivity disorder (ADHD) using measurement of event-related potentials during sustained attention as well as memory tasks are used as biomarkers to diagnose mild cognitive impairment [4–10].

Assessing and estimating the main indicators of attention, as well as simultaneous evaluation of the types of attention, have not been taken into account in previous research studies.

Therefore, if the main indicators of attention are estimated, various types of attention can be evaluated concurrently. In addition, determination of strength or weakness of any type of attention in a person to suggest a specific sports field, academic orientation, or a job has never been performed in the examined literature.

Despite numerous studies, no clear relationship between individual abilities in the field of attention and ERP brain signal has also been identified and proved so far.

Artificial intelligence has been described as the “fourth industrial revolution” that uses computers that can improve efficiency, accuracy, and precision similar to that of medical professionals in most cases [11].

Today, due to technological advances and demographic changes, diagnostic and treatment costs have become much more expensive [12] Also, time is an essential element in ensuring patient safety and performance, and time is a determining factor for patient satisfaction [13]. Therefore, machine learning and data mining should be used in order to optimize costs, time, and accuracy of diagnosis in health care.

In this research, the relationship between the electrical activity of the brain and types of attention has been identified and evaluated by extracting the features in the ERP brain graph. The noted graphs are utilized to measure the attention parameters simultaneously with the help of machine learning based on the IVA integrated visual and auditory test.

Therefore, a novel approach in improving the cognitive function of the brain is obtained based on the characteristics of brain electrical signals and neurofeedback.

## 2. Materials and Methods

### 2.1. IVA-CPT

Recently, IVA-CPT as a computerized test has been provided on the basis of DSM-IV criteria for assessing two universal scales (Scale Response Control and Scale Attention Quotient) [4, 14]. According to IVA-CPT, each type of attention originates from different parameters, which are mentioned as follows:

  Focused Attention: assessed primarily by the vigilance and prudence scales
  Sustained Attention: IVA + Plus scales related to this element are stamina, consistency, and focus
  Selective Attention: prudence, vigilance, and comprehension scales are reflective of this element.
  Divided Attention: relative differences in the prudence and speed scales may indicate problems or abilities for this element [15].

The process of sitting the test is as follows:

It is required for participants to click a mouse only when they see a “**3**” or hear a “**5**” (targets) and not to click when they see a “**5**” or hear a “**3**”(non-target).

The basic version of the main test consists of five quintiles of 100 trials each and, thus, the total test is comprised of 500 stimuli. Each trial lasts 1 second. The visual “**3**”s and “**5**”s are about 1.5 inches high on a VGA or higher resolution monitor and are presented for 220 milliseconds (ms). The presentation of the auditory “**3**”s and “**5**”s are for 500 ms, each. No feedback is provided during the main test.

### 2.2. EEG Recording

The ability to assess brain activity during performing a task has become a very important issue in the field of research and therapeutic diagnostics today. The event-related potential (ERP) recording device is utilized for monitoring brain function in a variety of situations.

The ERP recording system in the laboratory of the Islamic Azad University of Mashhad has been manufactured by MITSAR Company, which has been known as one of the leading manufacturers of EEG/ERP research devices in the world.

Nineteen Ag/AgCl electrodes connected to a portable brain amplifier with a sampling rate of 250 Hz and a 0.016–75 Hz band pass filter were used in this study to record the EEG signals. Electrodes (FP1, FP2, F7, F3, Fz, F4, F8, T3, C3, Cz, C4, T4, T5, P3, Pz, P4, T6, O1, and O2) were placed based on the International 10–20 system. Electrode AFz was considered as the ground and CPz was a common reference point. Prior to each session of recording, electrode impedances were measured. Furthermore, each channel's impedance was retained below 5 kΩ by the electrode gel. The electrodes were mounted on a wave guard cap.

### 2.3. Participants

The statistical population of the present study included 28 (22 males, 6 females) healthy individuals with different educational levels with average age of 27 ± 5.3 years old. They had no history in any types of mental or cognitive disorders. ERP recording as well as the IVA-AE2 test were performed during one session. However, the outcomes of the assessment were approved ultimately for 24 subjects. The protocol adopted in this research was in accordance with the Declaration of Helsinki, approved by the ethics committee of Azad University of Mashhad's Department of Biomedical Engineering. In addition, the research purpose was fully and clearly explained to all subjects and they were asked to provide their written informed consent to participate in the research.

### 2.4. Signal Preprocessing

After being amplified, the signals were then recorded in a computer by an analog-to-digital converter and analyzed by signal analysis software. Analysis of EEG data was carried out by the EEGLAB26 open source toolbox and custom MATLAB scripts (version R2016b, The MathWorks Inc.). The continuous data were first inspected visually and some parts of the EEG signal displaying marked as noise (consisting of channel disconnections) and they were manually discarded.

A finite impulse response (FIR) band-pass filter ranging from 1 to 30 Hz was next applied to the continuous data (filter order: 16500, −6 dB cut-off). Artifacts in the continuous EEG recording were recognized and removed using extended infomax-based independent component analysis (ICA).

Therefore, cognitive or sensory events, which were examined in ERP recording projects, were designed through the mentioned software.

This kind of software is designed to be synchronized with the brain signal display software and they show the timing of the stimulus on the Brainwave display, for analysis of relevant segments accurately. After initial processing, the segments were averaged and the final waveform was examined as a representation of the brain's response to the stimulus.

The special feature of ERP technique is high time accuracy that is not available in other neuroimaging techniques such as fMRI and PET scan. Brain activity can be monitored after providing a stimulus on a millisecond scale by using this technique. In many other neuroimaging methods, the noted scale is about 1.5 seconds.

**Figure 1:**
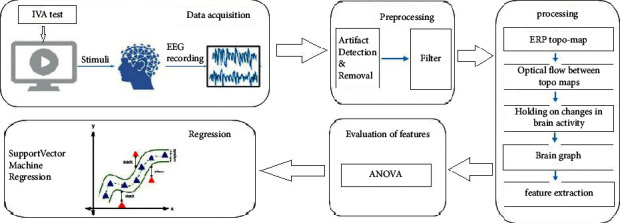
Flow chart of the proposed model.

The block diagram of the proposed method is depicted in Figure 1. The EEG signal is recorded in the first block. Preprocessing is performed in the second block and the main signal processing levels are carried out in the following blocks. Finally, a regression between the derived features and IVA test results is conducted in the SVR block.

### 2.5. EEG Signal Processing

Successively, the recorded ERP data were transformed into ERP topo-maps. In this article, changes in the activation between consecutive topo-maps were assessed using a differential computer vision-based method, known as Horn and Schunck. This optical flow assessment method was applied to position the flow of activation between different topo-maps [16].

The Horn and Schunck method aids to create the motion field between successive topo-maps, which is considered as the flow of activation between two time frames [16]. Motion vectors are then classified into different categories according to their activation levels. These clusters are chased between different frames as a measure of the activation flow. Finally, the activation flow across different brain lobes is investigated for different cases by plotting average activation graph with respect to time [16]. It is computed as follows:

(1) $$\begin{array}{c} u^{k+1}=u^{-k}-\frac{{\;I}_{x}\left({\;I}_{x}u^{-k}+I_{y}v^{-k}+I_{t}\right)}{4\alpha^{2}+I_{x}^{2}+I_{y}^{2}}\text{,}\\ v^{k+1}=v^{-k}-\frac{I_{y}\left({I_{x}u}^{-k}+I_{y}v^{-k}+I_{t}\right)}{4\alpha^{2}+I_{x}^{2}+I_{y}^{2}}\text{.} \end{array}$$

Here, *I*_*x*_, *I*_*y*_, and *I*_*t*_ are the derivatives of the image intensity values along the *x*, *y*, and time dimensions, respectively, $\overset{⟶}{V}={\left[u\left(x,y\right),v\left(x,y\right)\right]}^{T}$ , and the superscript *k* + 1 represents the next iteration, which is to be calculated, and *k* is the last calculated result.

In this study, the brain graph was obtained after acquiring the changes in the flow of brain activity in order to determine which features must be extracted.

It should be pointed out that some features must be extracted from the above graphs. The extracted features are listed in Table 1 [17].

After feature extraction, an SVR was used for regression between the derived features and IVA test results.

## 3. Results

**Figure 2:**
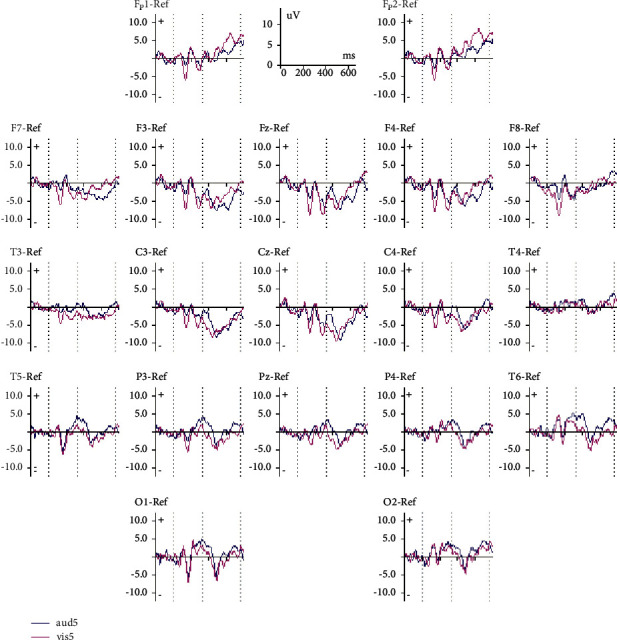
A grand averaged ERP recorded of auditory stimulation 5 (target) and visual stimulation 5 (non-target).

In the current study, ERP signal recording was performed on the subjects while performing the IVA test.  Figure 2 shows the extracted ERP for the target (auditory stimulation 5) and the non-target (visual stimulation 5). As it can be seen, there are differences in channels F7, F4, and P3.

**Figure 3:**
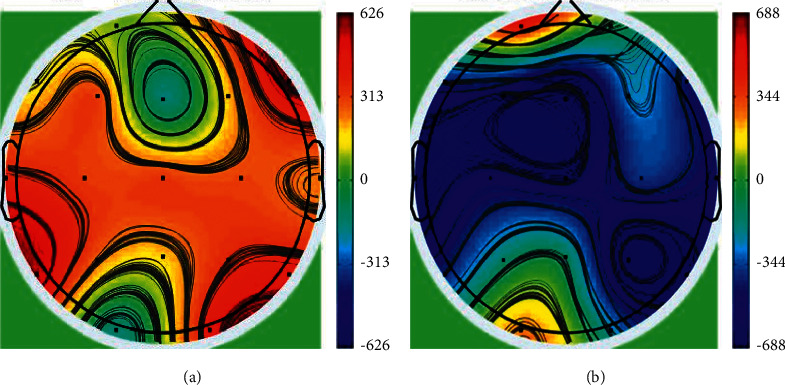
A sample of the extracted topo-map.

After recording the noted signals, brain maps were extracted from EEG/ERP data using EEGLab software, each of which indicates the activity status of each part of the brain in different frames. Sections with more active performance (which have been indicated in red) in the topo-maps are more important than other parts. In the current study, different sections of the map with high activity were identified. For this purpose, thresholds were set to specific areas with high activity. As colors of the regions move away from red to yellow, green, and blue, the activity of the brain in that specific part decreases. A sample of a brain map is indicated in Figure 3. The difference between two sub-figures indicates various patterns in brain maps. As it can be seen, the level of brain activity as well as the distribution of activity vary in different locations. For instance, the temporal region has a higher level of activity in Figure 3(a), while it is less active in Figure 3(b).

**Figure 4:**
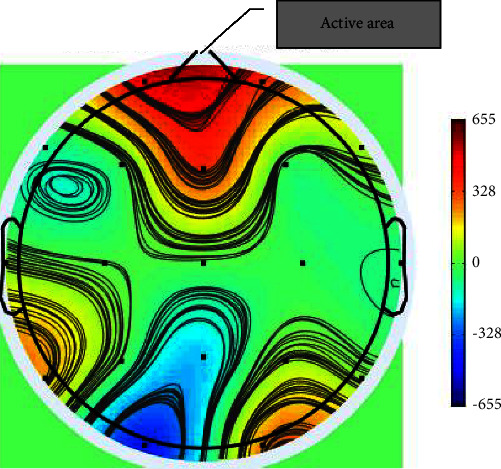
Identification of the active region of the brain.

In the next step, images were converted into grayscale. It should be noted that the threshold was set to grayscale = 150. Then, areas with high activity were identified in each frame as well as each map. As displayed in Figure 4, brain activity is above threshold in the frontal lobe which is known as an active area. Active area changes are noted in different frames.

Then, the optical flow method was applied on consecutive frame-based topo-maps to detect the flow of changes. In this way, motion vectors, which represented changes in the location of brain activity in different frames, were obtained. That is, how the active areas of the brain varied between different lobes of the brain during the test in each subject. In present research, an optical flow algorithm, namely HK, was implemented. The following figure indicates the output of mentioned technique.

**Figure 5:**
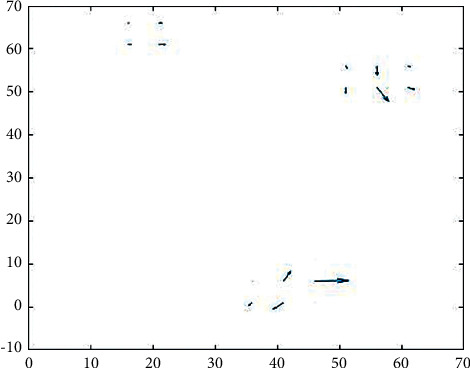
Change in two frames by applying the HK method.

Figure 5 shows the location, amount, and direction of information flow change in different topo-maps.

**Figure 6:**
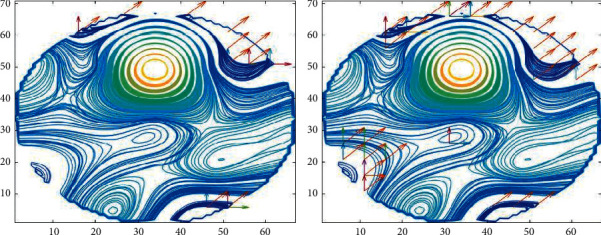
Samples of topo-maps and related changes.

Regarding Figure 6, it should be mentioned that the lower the threshold, the more active areas are considered in the threshold setting stage, the more motion vectors will definitely be observed which results in perplexing and misleading outcomes. Therefore, it was attempted to consider the highest threshold with trial and error to preserve the most information and minimize the number of vectors. After selecting the appropriate threshold limit, motion vectors for various frames were obtained. All these motion vectors were retained and plotted as a brain graph.

In the last stage, the ERP of each category of stimuli (visual target 3, auditory target 5, visual non-target 3, and auditory non-target 5) was divided into different frames. Then, two optical-flow methods were applied on all frames and a topo-map was created using the sum of motion vectors between different frames, a sample of which can be seen in Figure 6.

Figure 6 demonstrates the directional graph of the brain of a subject under special stimulation. As can be observed in the figure, changing the threshold led to some variations. Moreover, some parts were added to the graph. Therefore, choosing the precise threshold is very important. If the number of frames increases, the directionality of the motion vectors can be ignored and the threshold limit can be increased.

In the next step, features were extracted to quantify the brain graphs. The mentioned points are described in Table 1.

Finally, a regression between the extracted features and the IVA test outputs was performed with an SVR, the results of which are shown in Tables 2 and 3.

The leave one out (LOO) method was used in the SVR (with Gaussian kernel) for data division for training and testing.

The combination of parameters to achieve the amount of attention has been mentioned in the IVA guide section. A composition of two parameters, including vigilance and prudence, is obtained for focused attention. Noted parameters are used to examine the ability to stop reacting and not responding to the target. As the most important point in sustained attention is to maintain attention in time, all three parameters of stamina, consistency, and focus were applied to examine the speed of reaction and the response in different situations.

Due to the nature of selective attention, the comprehension parameter, which determines the amount of idiopathic errors is used in addition to vigilance and prudence parameters.

As attention is split in divided attention, two issues of the ability of non-automatic reaction and the speed of effective response are effective in this regard, so the parameters of prudence as well as speed have been implemented.

According to the abovementioned concepts, the high numerical value of each of the noted parameters shows a high rate of attention, thus, averaging was implemented to combine these parameters.

To estimate various types of attention according to the IVA clinical test, the basic indicators of attention should be combined according to Table 4 to obtain the amount of each type of attention. According to the results of Table 4, the proposed method has been more successful in estimating focused attention, but this does not mean that other estimates were not acceptable, but other attentions were estimated with acceptable accuracy. To clarify the issue, an example is provided. If the individual's scores on the IVA test are as follows, then the level of attention is gained based on Table 4.

Tables 5 and 6 shows a sample output of the IVA test in which (A) represents IVA test auditory and visual feature values and (B) indicates quantification of various types of attention through combining IVA test features. The important point in the present paper is that the IVA test indices were quantified with an accuracy of about 84.5% in the first step using the brain signal. Next, the level of attention was specified by combining these indicators. However, it is still emphasized that this method is associated with an error and cannot accurately indicate the level of attention. Nonetheless, it is very effective in tracking and detecting significant increase or decrease in the level of various types of attention in an online and simultaneous way.

## 4. Discussion

Assessment of attention plays a significant role in improving efficiency, protecting oneself from surrounding dangers, and diagnosing some psychological disorders such as anxiety, depression, and ADHD [18].

In clinical tests, although they have progressed and some of them have been computerized, human interventions reduce their validity and reliability. It is also not possible to perform these tests and measure attention during daily activities.

Due to the complexity and entanglement of different types of attention, it is difficult to separate them from each other. However, in the present proposed method, we were able to estimate the level of attention simultaneously for the first time which brings about two benefits as the following.

We can help improve people's performance and we can offer a more accurate treatment, for example, with neurofeedback, according to which type of attention and which indicators the person has more problems with.

A person may have a high level of focused attention but a low level of selective attention. With previous methods, it was not possible to measure all types of attention simultaneously. However, we can continuously monitor a person's attention level now and alert if there are significant changes.

Considering that “attention” is a complex cognitive index, features should be extracted to identify how the brain interacts and changes. Therefore, in this study, features were extracted from the brain graph that had the ability to track changes in brain activity in different lobes.

## 5. Conclusion

The EEG signal is maximally correlated with the measured behavior, regardless of the perceptual, cognitive, or motor process from which it is generated. In addition, since EEG-based devices can be portable, non-invasive, and have high temporal resolution technology for neural signal recording, they are very efficient [19].

The debate among the scientific communities about methods of assessing ADHD has been of interest for a long time. Therefore, it is necessary for researchers to continuously try to find the most suitable method to identify the cognitive and metacognitive deficits of people with hyperactivity [20].

Considering that the dominant symptom of ADHD is lack of attention, methods based on brain signals should be used to solve the limitations of existing clinical tests.

The proposed method has the ability to estimate the basic indicators of attention and simultaneously evaluate the types of attention, which is a unique study. However, it is suggested to increase the number of subjects, single test ERP, and design a game to replace the IVA test.

## Figures and Tables

**Table 1:** List of extracted features from topo-maps.

	Feature	Equation
1	Degree number of links connected to a node	*K*_*i*_=∑_*N*_*a*_*ij*_ Degree of a node i
2	Shortest path length, a basis for measurement of integration	*d*_*ij*_=∑_*N*_*a*_*uv*_ Shortest path length (distance), between nodes *i* and *j*,
3	Number of triangles, a basis for measuring segregation	*t*_*i*_=(1/2)∑_*N*_*a*_*ij*_*a*_*ih*_ *a*_*jh*_ Number of triangles around a node *i*
4	Measures of integration Characteristic path length	*L*=(1/*n*)∑_*N*_*L*_*i*_, where *L*_*i*_ is the average distance between node *i* and all other nodes.
5	Global efficiency	$E=\left(1/n\right)\underset{N}{\sum }E_{i}$ , where *ei* is the efficiency of node *i*.
6	Transitivity	*T*=∑_*N*_2*t*_*i*_/∑_*N*_*k*_*i*_(*k*_*i*_ − 1) Note that transitivity is not defined for individual nodes
7	Local efficiency	$E_{loc}=\left(1/n\right)\underset{N}{\sum }E_{loc.i},$ where *E*_loc_, *i* is the local efficiency of node *i*, and *djh* (*Ni*) is the length of the shortest path between *j* and *h*, that contains only neighbors of *i*.
8	Modularity	$Q=\underset{M}{\sum }\left[e_{uu}-{\left(\underset{M}{\sum }e_{uv}\right)}^{2}\right]$ , where the network is fully subdivided into a set of non-overlapping modules *M*, and *e*_*uv*_ is the proportion of all links that connect nodes in module *u* with nodes in module *v*.
9	Average neighbor degree	*k*_*nn*,*i*_=(∑_*N*_*a*_*ij*_*k*_*j*_/*k*_*i*_) Average degree of neighbors of node *i*

**Table 2:** Accuracy table based on features extracted from the target (HS method).

Parameter	Regression accuracy
Vigilance	73.6
Focus	83.5
Speed	76.9
Prudence	81.1
Consistency	83
Stamina	89.1
Comprehension	92

**Table 3:** Accuracy table based on features extracted from the non-target (HS method).

Parameter	Regression accuracy
Vigilance	86.3
Focus	61.5
Speed	43.2
Prudence	80.5
Consistency	73.5
Stamina	73.5
Comprehension	89.2

**Table 4:** Different types of attention and effective parameters.

Attention	Parameter	Accuraccy
Focused attention	(Vigilance + prudence)/2	**86.1**
Sustained attention	(Stamina + consistency + focus)/3	**83.4**
Selective attention	(Prudence + vigilance + comprehension)/3	**80.9**
Divided attention	(Prudence + speed)/2	**79.9**

**Table 5:** The amount of attention indicators.

	Aud	Vis
Vigilance	99	115
Focus	120	118
Speed	63	58
Prudence	108	114
Consistency	111	122
Stamina	103	107
Comprehension	110	108

**Table 6:** Amount of types of attention.

Attention	Aud	Vis
Focused attention	**104**/average	**115/**above average
Sustained attention	**112/**above average	**116/**above average
Selective attention	**106**/average	**113/**above average
Divided attention	**86/**slightly impaired	**86/**slightly impaired

## Data Availability

The datasets generated and analyzed during the current study are available from the corresponding author upon request (makhalilzadeh@mshdiau.ac.ir).
